# Prolonged Mechanical Stretch Initiates Intracellular Calcium Oscillations in Human Mesenchymal Stem Cells

**DOI:** 10.1371/journal.pone.0109378

**Published:** 2014-10-20

**Authors:** Tae-Jin Kim, Jie Sun, Shaoying Lu, Ying-Xin Qi, Yingxiao Wang

**Affiliations:** 1 Neuroscience Program, University of Illinois at Urbana-Champaign, Urbana, Illinois, United States of America; 2 Beckman Institute for Advanced Science and Technology, University of Illinois at Urbana-Champaign, Urbana, Illinois, United States of America; 3 Department of Bioengineering, University of Illinois at Urbana-Champaign, Urbana, Illinois, United States of America; 4 Department of Bioengineering and Institute of Engineering in Medicine, University of California San Diego, La Jolla, California, United States of America; 5 Institute of Mechanobiology and Medical Engineering, Shanghai Jiao Tong University, Shanghai, People’s Republic of China; The University of Akron, United States of America

## Abstract

Mesenchymal stem cells (MSCs) are a promising candidate for cell-based therapy in regenerative medicine. These stem cells can interact with their mechanical microenvironment to control their functions. External mechanical cues can be perceived and transmitted into intracellular calcium dynamics to regulate various cellular processes. Recent studies indicate that human MSCs (hMSCs) exhibit a heterogeneous nature with a subset of hMSCs lacking spontaneous calcium oscillations. In this study, we studied whether and how external mechanical tension can be applied to trigger and restore the intracellular calcium oscillation in these hMSCs lacking spontaneous activities. Utilizing the fluorescence resonance energy transfer (FRET) based calcium biosensor, we found that this subpopulation of hMSCs can respond to a prolonged mechanical stretch (PMS). Further results revealed that the triggering of calcium oscillations in these cells is dependent on the calcium influx across the plasma membrane, as well as on both cytoskeletal supports, myosin light chain kinase (MLCK)-driven actomyosin contractility, and phospholipase C (PLC) activity. Thus, our report confirmed that mechanical tension can govern the intracellular calcium oscillation in hMSCs, possibly via the control of the calcium permeability of channels at the plasma membrane. Our results also provide novel mechanistic insights into how hMSCs sense mechanical environment to regulate cellular functions.

## Introduction

Calcium signaling plays a pivotal role in regulating a wide range of cellular processes such as proliferation, differentiation, gene expression and cell death[1]–[3]. Over the past decades, intracellular calcium dynamics have been observed from many different cell types. Considerably various patterns of calcium oscillations or waves occur across different cell types and even within the same cell type, which were thought to be due to cell-specific calcium signaling proteomes [4], [5]. Although the complex spatiotemporal nature of intracellular calcium signaling is not yet fully understood, calcium oscillations arise partly due to periodic activation of calcium release from internal stores via inositol 1,4,5-trisphosphate receptors (IP_3_Rs) and ryanodine receptors [6].

Previous studies suggest that intracellular calcium signaling is closely interconnected with mechanical properties of a cell. For instance, at the plasma membrane, mechanosensitive calcium permeable channels such as transient receptor potential (TRP) channels that generate changes in intracellular calcium concentration are activated upon various mechanical stimuli[7]–[9]. Accumulating evidences show that the activation of mechanosensitive calcium permeable channels depends on elastic properties of the extracellular matrix (ECM) surrounding cells, and a complex interplay between actin cytoskeleton and cell adhesion sites [10], [11]. Therefore, the integrated mechanical property of a cell could be one of the potential factors in the regulation of intracellular calcium dynamics, such as calcium oscillations, upon mechanical stimulation. Although calcium dynamics have been extensively studied in many ways, it still remains unclear how cells perceive the mechanical cues to regulate calcium oscillations.

Some human mesenchymal stem cells (hMSCs) exhibit spontaneous calcium oscillations, while others do not [10], [12], [13]. The exact reason of this heterogeneous nature remains unclear, although it is possible that the heterogeneous mechanical tension in individual stem cells may result in different calcium permeability of membrane channels which serve as the trigger of intracellular calcium oscillations via calcium influx across the plasma membrane and calcium induced calcium release [10]. In this study, we focus on the subpopulation of hMSCs lacking calcium oscillations to study how the mechanical cues can trigger the intracellular calcium oscillations and regulate calcium dynamics. To perform this study, we took advantage of the fluorescence energy resonance transfer (FRET)-based calcium biosensor to monitor the intracellular calcium dynamics, which enables us to visualize the dynamics with high spatiotemporal resolutions in live hMSCs. Our results demonstrate that prolonged mechanical stretch (PMS) initiates intracellular calcium oscillations in this subpopulation of hMSCs, mediated by the cytoskeletal support, actomyosin contractility and phospholipase C (PLC) activity. Thus, our report helps to advance our understanding on how hMSCs perceive external mechanical environment to regulate intracellular molecular signals.

## Materials and Methods

### Cell culture, transfection and chemicals

Human mesenchymal stem cells (hMSCs; Lonza Walkersvile, Inc., Walkersvile, MD) were maintained in mesenchymal stem cell growth medium (MSCGM, PT-3001, Lonza) containing 10% fetal bovine serum (FBS), 2 mM L-glutamine, 100 U/ml penicillin and 100 µg/ml streptomycin in a humidified incubator of 95% O_2_ and 5% CO_2_ at 37 °C. Streptomycin was not included in the medium during imaging experiment because it is known as an inhibitor of stretch-activated channels. The DNA plasmids were transfected into the cells using Lipofectamine 2000 (Invitrogen, Carlsbad, CA) reagent according to the product instructions. Gadolinium chloride (GdCl_3_), 1,2-bis(o-aminophenoxy)ethane-N,N,N',N'-tetraacetic acid (BAPTA), Cytochalasin D, Nocodazole, ML-7, U73122, Nifedipine and 2-Amino-ethoxydiphenylborate (2-APB) were purchased from Sigma Aldrich (St. Louis, MO). Neomycin sulfate was purchased from Santa Cruz Biotechnology (Santa Cruz, CA).

### Genetically encoded FRET biosensors

The construct of FRET-based calcium biosensor has been well described in previous studies [10], [14]. In order to enhance the FRET efficiency, the FRET pair was replaced by enhanced fluorescent protein (ECFP) and an improved version of yellow fluorescent protein (YPet). Briefly, the fragment containing ECFP, Calmodulin (CaM)-M13 peptide, was fused to YPet, and subcloned into pcDNA 3.1 (Invitrogen) for mammalian cell expression using BamHI and EcoRI sites.

### Bis-acrylamide polyacrylamide gel fabrication

Polyacrylamide (PA) gels were prepared on aminosilanized glass cover slips as previously described [14]. Briefly, 40% w/v acrylamide and 2% w/v bis-acrylamide stock solutions (Bio-Rad, Hercules, CA) were mixed to prepare a PA solution and then the gel’s stiffness was achieved by varying the final concentration of the PA solution (3% ∼7.5%) and Bis-acrylamide cross-linker (0.06% ∼0.4%). To polymerize the solutions, 2.5 µl of 10% w/v ammonium persulfate (APS; Bio-Rad, Hercules, CA) and 0.25 µl of N,N,N′,N′-Tetramethylethylenediamine (TEMED; Bio-Rad, Hercules, CA) were added to yield a final volume of 500 µl PA solution. To crosslink extracellular matrix molecules onto the gel surface, a photoactivating cross-linker, 0.5 mg/ml of sulfo-SANPAH (sulfosuccinimidyl 6-(4′-azide-2′-nitrophenyl-amino) hexanoate, Pierce, Rockford, IL) solution was used. The powder of sulfo-SANPAH was dissolved in 10 mM HEPES buffer containing 0.5% DMSO and the solution was added on top of the PA gel. Gel dishes were placed at a distance of ∼15 cm from the UV light of the hood for 6 min and rinsed three times with 10 mM HEPES buffer for 10 min. A 200 µl of fibronectin solution (5, 10 or 40 µg/ml) from bovine plasma (Sigma, St. Louis, MO) was incubated on top of the sulfo-SANPAH-coated PA gel at 37°C overnight to deposit fibronectin for subsequent cell seeding.

### Mechanical stretch equipment

The mechanical stretch equipment was described in our previous study performed by Dr. Nishitani [14]. In brief, this device was designed to use a glass capillary probe tip to poke and pull the PA gel substrate (20 kPa) on which cells were seeded. This tip was precisely positioned using two sets of XYZ linear stages (Newport MT-XYZ model, Newport 461 Series) 15 −20 µm away from the cell edge with 25 µm depth of penetration into the gel substrate. Pulling the tip parallel to the gel applied tangential stretch to the gel substrate. The strain map was calculated in the substrate where cells were seeded. The position of the beads in the substrate between two frames (a frame; PMS loading, a reference frame: unloading) were analyzed, and both the displacement/strain index maps were reconstructed by a customized Matlab (MathWorks).

The total strain, E was calculated as:
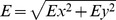
where *E_x_* and *E_y_* are the strain in x and y axis.



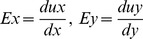
where *ux* and *uy* are the displacements in x and y axis. The measured peak strain of substrate occurring at the position of the cell was approximately 0.3. The peak stress (strain x Young’s modulus) applied in this study was ∼6000 N/m^2^. When the cells were subjected to the mechanical stretch, the strain value was measured up to ∼0.3. The cells were exposed on 20 kPa (20000 N/m^2^) gel substrate. Hence, the total stress (S) was calculated as S = Strain x Young’s modulus. S = 0.3 × 20000 N/m^2^ = 6000 N/m^2^. During live-cell imaging, the cells were maintained in CO_2_-independent medium (Invitrogen) containing 0.5% FBS and the temperature of 37 °C was kept by a controlled heater (Nevtek ASI 400).

### Live-cell FRET imaging

Images were collected by a Zeiss Axiovert microscope 200 equipped with a cooled CCD (Charge-Coupled Device) camera and controlled by MetaFluor 6.2 software (Molecular Devices). For the FRET imaging, a 420DF20 nm excitation filter and two emission filters (475DF40 nm for ECFP and 535DF25 for YPet) were used to collect the emissions. The cells were imaged to record the emission ratio of FRET and then the imaging acquisition was paused to place the glass tip probe in position on the PA gel and resumed after the mechanical pulling was applied. The image files were analyzed by MetaFluor 6.2 software (Molecular Devices) and Microsoft Excel.

### Data analysis

The frequency of calcium oscillations was calculated as the average number of oscillation per minute and also amplitude (calcium peak) was calculated as the average peak of the FRET/ECFP emission ratio. Comparisons between two groups were analyzed using Student’s t-test in Excel. The values were presented as the means ± standard deviation (SD). Significant differences were determined by the p-value (<0.05). For comparisons between multiple groups, we used the Bonferroni multiple comparison test of means at 95% confidence interval, which is provided by the multicompare function in the MATLAB statistics toolbox (The MathWorks, Natick, MA).

## Results

### Chemical and mechanical characterization of the spontaneous calcium oscillations in hMSCs

**Figure 1:**
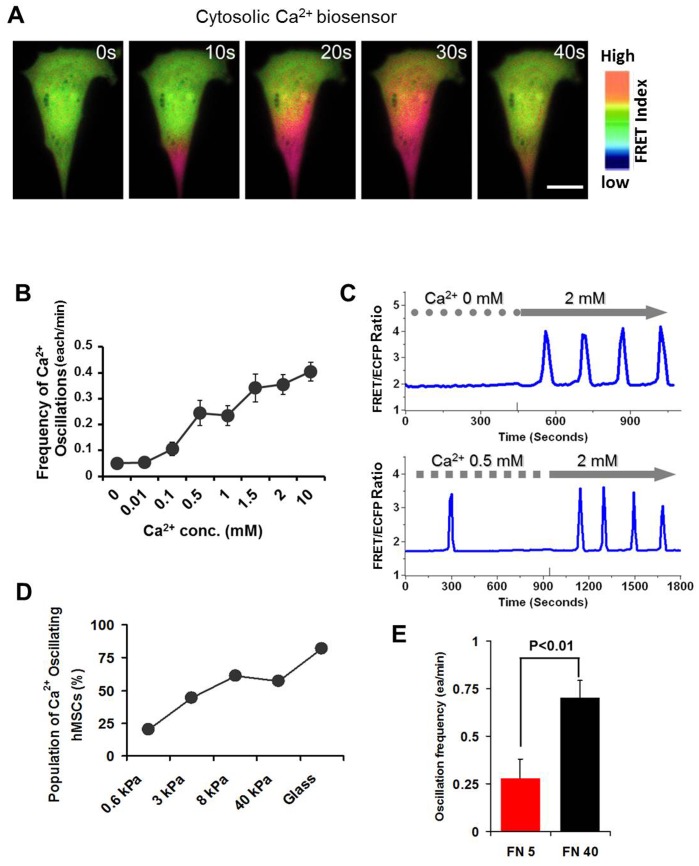
Calcium oscillations depend on the extracellular calcium concentration and mechanical microenvironment. (A) Cytosolic FRET-based calcium sensor shows the intracellular calcium dynamics with high spatiotemporal resolutions in hMSC. Red and blue colors represent the high and low FRET ratios, respectively. Images were captured in 10 second interval. Scale bar, 20 µm. (B) Frequency of calcium oscillations (the number of peaks per minute) is measured in cells subjected to various extracellular calcium concentrations (0 mM to 10 mM, n = 14–32). (C) Time courses of calcium signals as imaging medium is replaced with physiological calcium concentration (2 mM). (D) Estimated percentage of hMSCs showing spontaneous calcium oscillations on different substrates. (n = 18–33). (E) Bar graphs show that the strength of Integrin-mediated cell adhesion with increased fibronectin (FN) concentration affects the frequency of calcium oscillations. The frequency of calcium oscillations on FN 40 (40 µg/ml)-coated gel are significantly higher than that from FN 5 (5 µg/ml) (**P<0.01, n = 7).

To examine whether the spontaneous calcium oscillations can be triggered by external physiological and mechanical conditions, we have utilized FRET based calcium biosensor to visualize and monitor intracellular calcium signals with high spatiotemporal resolutions in live hMSCs. As shown in Figure 1A, some hMSCs transfected with cytosolic calcium biosensor have shown intracellular calcium dynamics, with red color in images representing the high FRET/ECFP ratio and hence high intracellular calcium concentration. Using the cytosolic calcium sensor, we can evaluate the frequency of calcium oscillations under various extracellular calcium concentrations (Fig. 1B and 1C). We observed that the frequency of spontaneous calcium oscillations was dependent on the extracellular calcium concentration. At physiological (2 mM) and higher calcium concentrations (10 mM), cells showed a higher frequency of oscillations compared to cells cultured with lower calcium concentrations (0 −1 mM) (Fig. 1B). Increase of calcium concentration in the medium enhanced this frequency (Fig. 1C). In addition to these calcium conditions, when the cells were exposed to different substrate stiffness (Young’s E-modulus), the frequency of oscillations increased with increasing substrate stiffness. As shown in Figure 1D, a large population of cells cultured on glass bottom dishes (approximately 81%) showed spontaneous calcium oscillations, which is consistent with the previous observations [10], [12], [13]. In contrast, in cells cultured on soft substrate (0.6 kPa), only approximately 20% of cell population showed the spontaneous calcium oscillations. Consistently, the enhancement of integrin-mediated cell adhesion on hard gel substrate (40 kPa) with high fibronectin (FN) concentration (40 µg/ml) has markedly increased the frequency of spontaneous calcium oscillations compared to cells on low FN concentration (5 µg/ml) (Fig. 1E). Taken together, these results suggest that the external calcium concentration, the concentration of extracellular matrix protein and the stiffness of substrate all affect the spontaneous calcium oscillation in hMSCs.

### Restoration of calcium oscillations by PMS

**Figure 2:**
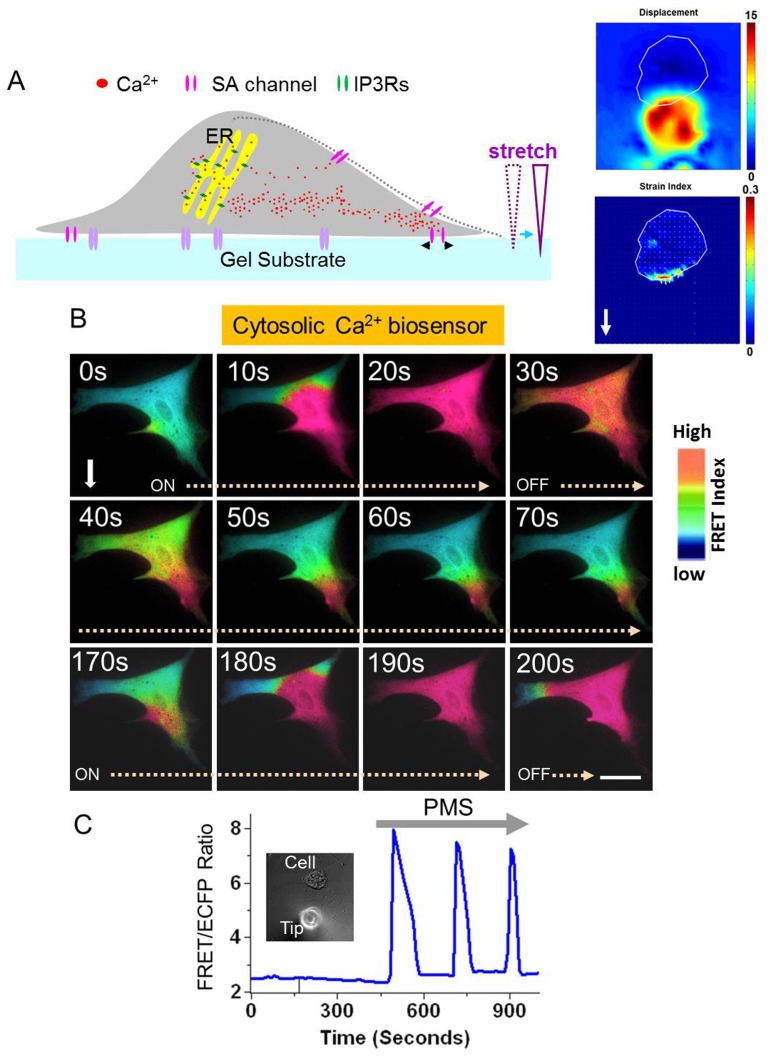
Prolonged mechanical stretch (PMS) triggers the calcium oscillations in hMSCs without spontaneous activities. (A) A schematic drawing of PMS application. Cells were seeded on polyacrylamide (PA) gels with 20 kPa of elastic modulus and a glass capillary probe tip can be placed near a cell and inserted in the gel to pull the gel and cause the stretch of the target cell. The right images show typical displacement and strain maps during prolonged mechanical stretch. The cold and hot colors represent the small and large relative displacement (top, 0–15 µm) and strain index (bottom, 0–0.3). (B) Mechanical stretch was applied to the cytosolic FRET biosensor-transfected hMSC and intracellular calcium dynamics were visualized by the FRET ratio. High FRET ratio (Red color) denotes high concentration of calcium. White arrow points to the direction of mechanical stretch. ON: calcium increase, OFF: calcium decrease. Scale bar, 20 µm. (C) The time course shows that PMS triggers the calcium oscillations in a cell without spontaneous activities. DIC image shows the cell and the probe tip on the PA gel.

Since it is reported that a subpopulation of hMSCs has no spontaneous calcium oscillations, we have chosen this group of cells to examine whether calcium oscillations can be triggered by external mechanical stimulation. To perform this experiment, we applied the mechanical stretch equipment that has been developed previously in our laboratory [14]. This equipment was designed to insert a glass probe tip into the gel substrate and pull the gel near cells, and subsequently induce a deformation and elongation of the target cell by prolonged stretch. Figure 2A (left) shows a schematic drawing of this system and the typical maps of displacement and strain (right), with the cold and hot colors representing the small and large changes in displacement (top image) and strain (bottom image), respectively. Upon stretch, the cell is expected to expand in the direction of pulling, which will trigger the calcium permeable membrane channels. Our FRET imaging showed that PMS triggered local calcium elevation at a region proximal to the probe location, possibly through the activation of stretch-activated calcium permeable channels (Fig. 2B). Indeed, calcium influx is necessary for PMS-induced calcium oscillation in hMSCs as elimination of extracellular calcium totally abolished PMS-induced calcium oscillations (Fig. S1A). In addition, the mechanical stretch has to be prolonged as a transient stretch is not able to sustain calcium oscillations (Fig. S2). More interestingly, the local calcium elevation propagated to the distal region of the cell and eventually induced the calcium oscillations. This pattern of calcium oscillations triggered by mechanical stretch is similar to the spontaneous calcium oscillations in hMSCs (Fig. 2B–C and Fig. 1C). Thus, our results suggest that the calcium oscillations can be triggered by PMS in these hMSCs without spontaneous calcium activities.

### Triggered calcium oscillations by PMS rely on calcium influx via stretch-activated calcium permeable channels

**Figure 3:**
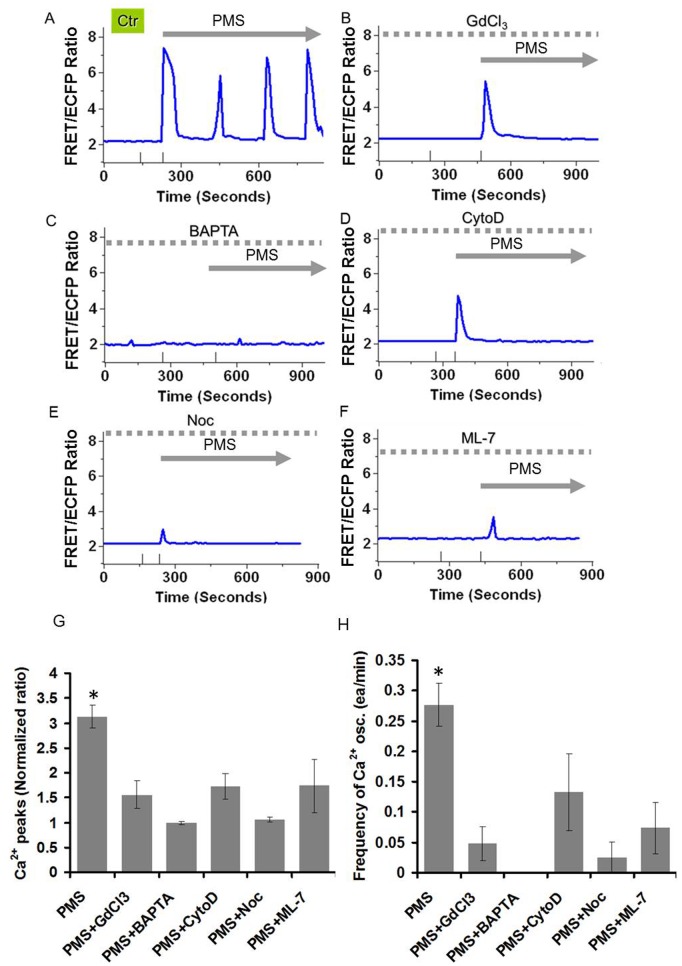
Calcium oscillations triggered by the PMS depend on calcium entry via the plasma membrane, cytoskeleton and actomyosin contractility. (A) PMS evokes calcium oscillations in a subpopulation of hMSCs without spontaneous activities (Ctr; control). (B, C) PMS-induced calcium oscillations were inhibited in GdCl_3_- or BAPTA-pretreated cells, suggesting the involvement of calcium entry at the plasma membrane. (D, E, F) PMS-induced calcium oscillations were also inhibited in CytoD-, Noc-, or ML-7 pretreated cells, suggesting the involvement of cytoskeleton and actomyosin contractility in the PMS-induced calcium oscillations. Amplitude (G) and frequency (H) of PMS-induced calcium oscillations are inhibited in the presence of inhibitors. PMS, control group with prolonged mechanical stretch only (n = 9); PMS+GdCl_3_ (5 µM), an inhibitor of stretch-activated calcium channel was applied 1 hr before stretch (n = 4); PMS+BAPTA (20 µM in the calcium free medium), a calcium chelator was applied before stretch (n = 5); PMS+CytoD (1 µM), an inhibitor of actin filament was applied 1 hr before stretch (n = 5); PMS+Noc (5 µM), an inhibitor of microtubule was applied 1 hr before stretch (n = 4); PMS+ML-7 (10 µM), an inhibitor of MLCK was applied 1 hr before stretch (n = 4). * indicates statistically significant difference from all other groups by the Bonferroni multiple comparison test with p<0.05.

To examine whether calcium influx from the extracellular environment via stretch-activated calcium permeable channels is involved in the calcium oscillations triggered by PMS, we used a potent inhibitor of stretch-activated calcium permeable channels, Gadolinium chloride (GdCl_3_, 5 µM) or a calcium chelator, 1,2-bis(o-aminophenoxy)ethane-N,N,N′,N′-tetraacetic acid (BAPTA, 20 µM). As shown in Figure 3B–C, both GdCl_3_ and BAPTA inhibited the mechanical stretch-induced calcium oscillations compared to the control group (Fig. 3A). Indeed, both amplitude and frequency of calcium oscillations have been significantly reduced in these two groups with inhibitor treatment (Fig. 3G–H). We also tested the effect of 2-APB (50 µM), an inhibitor of TRP channels, and Nifedipine (10 µM), an inhibitor of L-type Ca^2+^ channel on the PMS-induced calcium oscillations. We observed that the PMS induced calcium signal was abolished in 2-APB-treated cells, whereas Nifedipine had no effect (Fig. S1C–D). Taken together, our results suggest that calcium entry via stretch-activated calcium permeable channels from the extracellular environment upon mechanical stretch is essential for the stretch-induced calcium oscillations in hMSCs.

### Cytoskeleton and MLCK-driven actomyosin contractility mediate calcium oscillations induced by PMS

Since cytoskeletal dynamics and MLCK-driven actomyosin contractility have a significant impact on the cellular tension, we have examined whether they can contribute to the induction of calcium oscillations in response to PMS. Either Cytochalasin D (CytoD, 1 µM) or Nocodazole (Noc, 5 µM) treatment suppressed the PMS-induced calcium oscillations (Fig. 3D–E). Both amplitude and frequency of calcium oscillations were significantly inhibited by the treatment of these inhibitors, suggesting that intracellular cytoskeletal support through actin filaments and microtubules are necessary for the induction of tension-dependent calcium oscillations (Fig. 3G–H). Consistently, ML-7 (10 µM), an inhibitor of MLCK, significantly attenuated the stretch-induced calcium oscillations in comparison to control cells (Fig. 3F–H). Therefore, these results suggest that both passive cytoskeletal supports and active MLCK-driven actomyosin contractility are essential for the triggering of calcium oscillations by prolonged mechanical stretch.

### PLC/IP_3_ contributes to prolonged mechanical stretch-induced calcium oscillations

**Figure 4:**
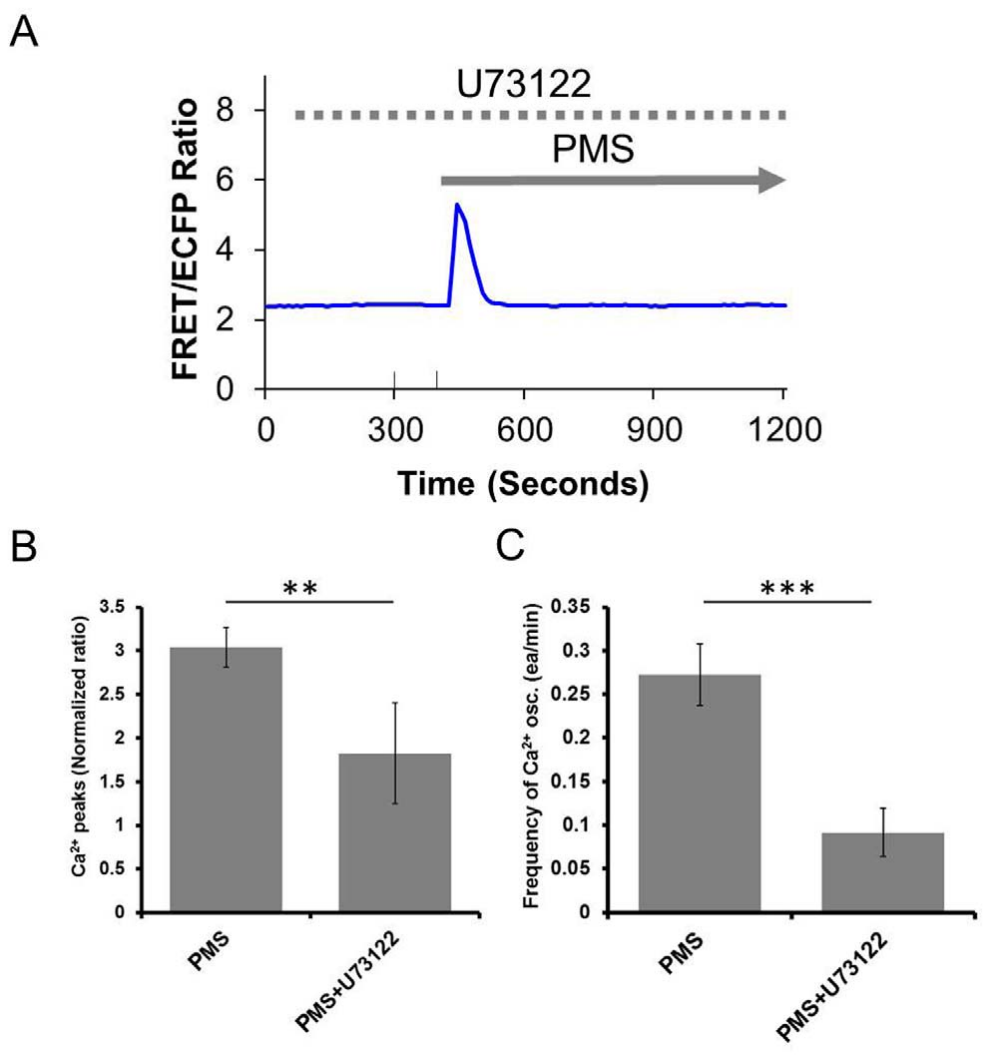
The effect of PLC inhibitor on the PMS-induced calcium oscillations. (A) In U73122 (5 µM, 30 min) pretreated hMSCs, PMS does not induce sustainable calcium oscillations. (B–C) Both amplitude and frequency of PMS-calcium oscillations are inhibited in the presence of U73122 (PMS, n = 4; PMS+U73122, n = 4, **P<0.01 and ***P<0.001).

PLC plays an important role in the regulation of intracellular calcium concentration [15], so we examined whether PLC can contribute to the generation of the stretch-induced calcium oscillations. In the presence of PLC inhibitor, U73122 (5 µM), calcium oscillations were markedly suppressed in response to PMS (Fig. 4). Another type of PLC inhibitor, Neomycin (10 µM), also inhibited the PMS-induced calcium oscillations (Fig. S1B). As 2-APB (50 µM) is also an inhibitor of IP_3_Rs, our observation that the PMS induced calcium signal was abolished in 2-APB-treated cells supports the notion that the PMS-induced calcium oscillations depend on the PLC/IP_3_ signal transduction and its subsequent calcium release from internal calcium store (Fig. S1C). Our data is hence consistent with the previous observation that PLC remains inactive at basal intracellular calcium concentrations, but become activated when the intracellular calcium concentration rises [15], possibly from the stretch-activated channel activities at the plasma membrane. This result is similar to our previous data that disruption of PLC/IP_3_ generation inhibited the intracellular calcium increase induced by mechanical vibration in HUVECs [14].

## Discussion

**Figure 5:**
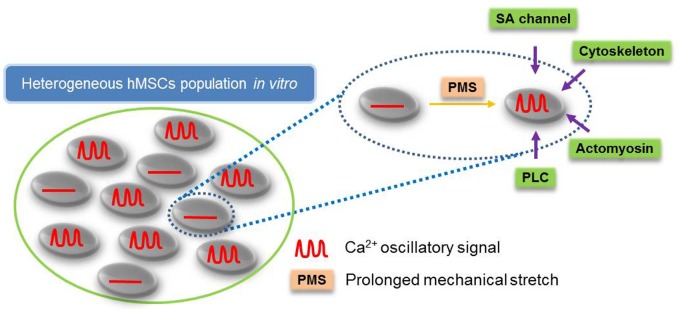
PMS-induced calcium oscillation in a subpopulation of hMSCs lacking the spontaneous calcium oscillation. A schematic drawing represents that PMS-induced calcium oscillations depend on stretch-activated channels, cytoskeletal supports, actomyosin contractility and PLC activity.

The importance of mechanical cues to mesenchymal stem cell functions has been well established [16]. Our previous study showed the frequency and amplitude of the oscillation increases with increasing substrate stiffness [10] while our current study showed the population of cells with spontaneous calcium oscillation also increases (Fig. 1D), confirming the significant effect of mechanical environment on calcium signaling in hMSCs. However, studies of substrate rigidity mostly revealed the equilibrated states of cells after their long-time adaptation to different mechanical environments while PMS as an acute mechanical stimulation can deliver an active mechanical impact. To clearly examine the effect of this acute mechanical stretch on intracellular calcium dynamics in hMSCs, we focus on the naturally-occurring inactive population of hMSCs that lack spontaneous oscillation, which provides a model where the exact effect of PMS can be tested on a minimal background (Fig. 5). It would also be interesting to study the effect of this acute mechanical stimulation on the active population of hMSCs in the future and compare the responses from different populations, although there is a possibility that the calcium oscillation machinery is already saturated in these spontaneous oscillating cells which can mask the effect of mechanical stimulation.

Our results not only showed the effectiveness and sufficiency of PMS on initiation of calcium oscillation in inactive population of hMSCs but also elucidated the possible molecular mechanisms of PMS’s effect. The observation that extracellular calcium, stretch-activated calcium permeable channels, passive cytoskeleton support and active actomyosin are all needed for PMS-induced oscillation suggest that PMS may initiate calcium oscillation by calcium influx through the calcium permeable channels at the plasma membrane, which are coupled to cytoskeletal structures (Fig. 5) [10], [17]. Notably, the continuous application of mechanical load in our system would not lead to constant increase in intracellular calcium concentration. This is consistent with the hypothesis that the mechanical-induced calcium signals are encoded in the frequency, but not amplitude, of calcium oscillations [18]. The disappearance of the oscillations upon removal of PMS suggests that prolonged stretch is needed to sustain the calcium oscillations (Fig. S2). In addition, GdCl_3_ inhibited the sustained calcium oscillation even though the first peak was still there (Fig. 3B), suggesting that stretch-activated channels are needed to sustain the calcium oscillations. Notable, the first calcium peak may not entirely depend on stretch-activated cation channels, as the initial calcium peak induced by PMS is resistant to related inhibitors (Fig. 3). It is possible that the initial application of PMS causes a sudden calcium influx across the plasma membrane via stretch-activated channels and other nonspecific transient leakage to result in the first peak of calcium signals. As cells adapt to the sustained PMS, the nonspecific leakage may disappear therefore the subsequent oscillations depend only on stretch-activated channels, which can be abolished by GdCl_3_. In addition to the calcium signal originated from the direct activation of stretch-activated channels, it is also possible that PMS activates integrin receptor which in turn activates IP_3_Rs. However, this may occur after the activation of stretch-activated channels. Indeed, Ingber group showed the ultra-rapid activation of mechanosensitive calcium permeable channels, Transient Receptor Potential Vanilloid-4 (TRPV4), can allow calcium entry within 4 ms of mechanical stimulation [19]. In contrast, integrins are only activated minutes after mechanical stimulation [20], [21].

The comparison of the PMS-induced calcium oscillations to spontaneous ones in hMSCs shows several differences and similarities, possibly reflecting a complex and coordinated nature of calcium regulation in hMSCs. First, Nifedipine has no effect on PMS-induced calcium oscillations while it completely blocks spontaneous ones in our previous study [10] (Fig. S1D). As Nifedipine is a blocker for L-type Ca^2+^ channels, L-type Ca^2+^ channel is needed for spontaneous calcium oscillations but may be dispensable for PMS-induced ones. Second, the spontaneous calcium oscillations were not abolished by either inhibition of cytoskeleton or MLCK-driven actomyosin contractility [10], while PMS-mediated calcium oscillations depend on both. This suggests that the intracellular mechanical support and tension are needed for the PMS-induced oscillation but not for the spontaneous ones. Despite these listed differences, there are some similarities as both stretch-activated calcium permeable channels at the plasma membrane and the IP_3_Rs at the ER membrane are essential for these two modes of oscillations. In addition, the frequency of calcium oscillation in PMS-induced oscillations was not statistically different from that of spontaneous ones (Fig. S3), suggesting that they may share some common players for calcium regulation.

The family of stretch-activated calcium permeable channels is essential for both spontaneous and PMS-induced oscillations. While the exact molecular mechanism remains unclear, several candidates may be involved. For example, Transient Receptor Potential Melastatin 7 (TRPM7) plays an important role in not only actomyosin-mediated contractility in response to mechanical forces via phosphorylation of myosin-II heavy chain, but also cell adhesion by inducing the transformation of focal adhesions [22]. Activation of calcium influx via TRPV4 channels in response to mechanical force can also be mediated by β1 integrins, transmembrane receptors that bridge for ECM adhesions [19]. In addition, TRPV4 agonist-induced calcium entry can be directly associated with actomyosin network [23]. Transient Receptor Potential Channel 1 (TRPC1) and Transient Receptor Potential Channel 6 (TRPC6) expressed in MSCs [24] might also be cooperating with TRPV4 channels in mediating intracellular calcium signaling in response to mechanical stimuli [25].

Characterizing the response of the inactive cells to PMS not only helps us identify the direct and dynamic mechanical regulation of the calcium signaling but also provides information to understand the difference between the active and the inactive populations for future studies. A differential expression of functional ion channels between spontaneously active and inactive cells may contribute to these distinctive features of calcium regulations. In fact, Nifedipine-sensitive L-type Ca^2+^ currents are rather heterogeneous in subpopulations of rat MSCs and human MSCs [26], [27]. It is hence possible that the spontaneously active population is enriched with Nifedipine-sensitive L-type Ca^2+^ channels, which have relatively low expression within the inactive population. Another difference could be engendered from heterogeneous mechanical properties of the cells [28]. As substrate rigidity can change the mechanical properties of the cells [29], the regulation of the calcium oscillations by substrate stiffness suggests that mechanical properties of the cells can affect their calcium signaling [10]. Notably, the differential expression of membrane proteins/channels and mechanical properties of cells are not isolated events. For example, cellular tension has a profound effect on gene expression and lineage specification while the differential expression of adhesion as well as cytoskeletal molecules can affect the mechanical properties of cells [30], [31]. Last but not least, the partition between inactive and active populations is not static as the percentage of cell population with spontaneous calcium oscillation increases with increasing substrate rigidities (Fig. 1D). It is not inconceivable then that cells can undergo transition between these two states. Further research on the differences between the inactive and active populations of hMSCs should shed new light on the chemical and mechanical regulation of hMSCs functions.

In summary, our calcium FRET biosensor clearly allows the observation of intracellular calcium dynamics with high spatiotemporal resolutions in hMSCs under different microenvironments. PMS triggered intracellular calcium oscillations in a subset of hMSCs lacking spontaneous calcium activities (Fig. 5). These stretch-induced calcium oscillations are dependent on the mechanical support of cytoskeleton and MLCK-driven actomyosin network. A proper PLC activity also contributes to the generation of calcium oscillations induced by PMS. Thus, our results provide novel information to advance our understanding on the underlying molecular mechanisms by which hMSCs perceive external mechanical environment to regulate intracellular molecular signals and potentially pathophysiological consequences.

## Supporting Information

Figure S1 The PMS-induced calcium oscillations in response to (A) the Ca^2+^ -free condition (0.5 mM EGTA in the absence of CaCl_2_, n = 3) and pharmacological inhibitors, (B) 2-APB (50 µM, n = 3), an inhibitor of IP_3_Rs and TRP channels, (C) Nifedipine (10 µM, n = 3, no statistical difference), an inhibitor of L-type Ca^2+^ channels, (D) Neomycin (10 µM, n = 3), an inhibitor of PLC. (E) Each group (A–C) was compared against PMS control and Student t-test was performed. (* P<0.05, ** P<0.01.)
(TIF)

Figure S2 Calcium signals in response to PMS and unloading. hMSCs showed the PMS-induced calcium signals, but when stretch stopped, calcium oscillations disappeared (n = 3, Loading vs Unloading,** P<0.01).
(TIF)

Figure S3 Spontaneous calcium oscillations (n = 33) have similar frequency as PMS-induced calcium oscillations (n = 9). There is no statistical difference.
(TIF)
