# Supplementary figures and images for: Prolonged Mechanical Stretch Initiates Intracellular Calcium Oscillations in Human Mesenchymal Stem Cells
Source: PLoS One. 2014 Oct 20;9(10):e109378. doi: 10.1371/journal.pone.0109378 (PMC4203723; doi:10.1371/journal.pone.0109378)

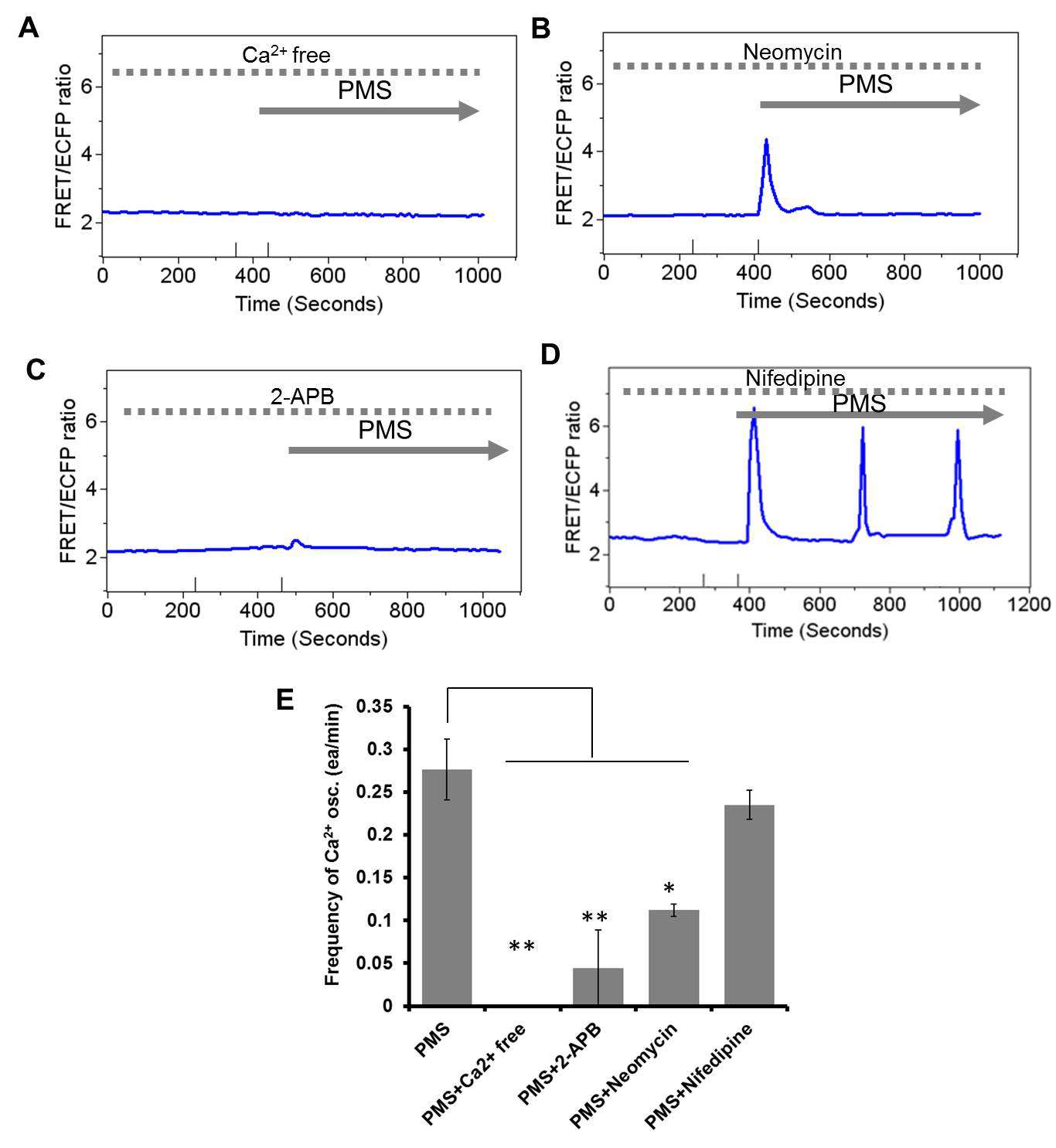

Supplement: Figure S1 — The PMS-induced calcium oscillations in response to (A) the Ca2+ -free condition (0.5 mM EGTA in the absence of CaCl2, n = 3) and pharmacological inhibitors, (B) 2-APB (50 µM, n = 3), an inhibitor of IP3Rs and TRP channels, (C) Nifedipine (10 µM, n = 3, no statistical difference), an inhibitor of L-type Ca2+ channels, (D) Neomycin (10 µM, n = 3), an inhibitor of PLC. (E) Each group (A–C) was compared against PMS control and Student t-test was performed. (* P<0.05, ** P<0.01.) (TIF) [file pone.0109378.s001.tif]

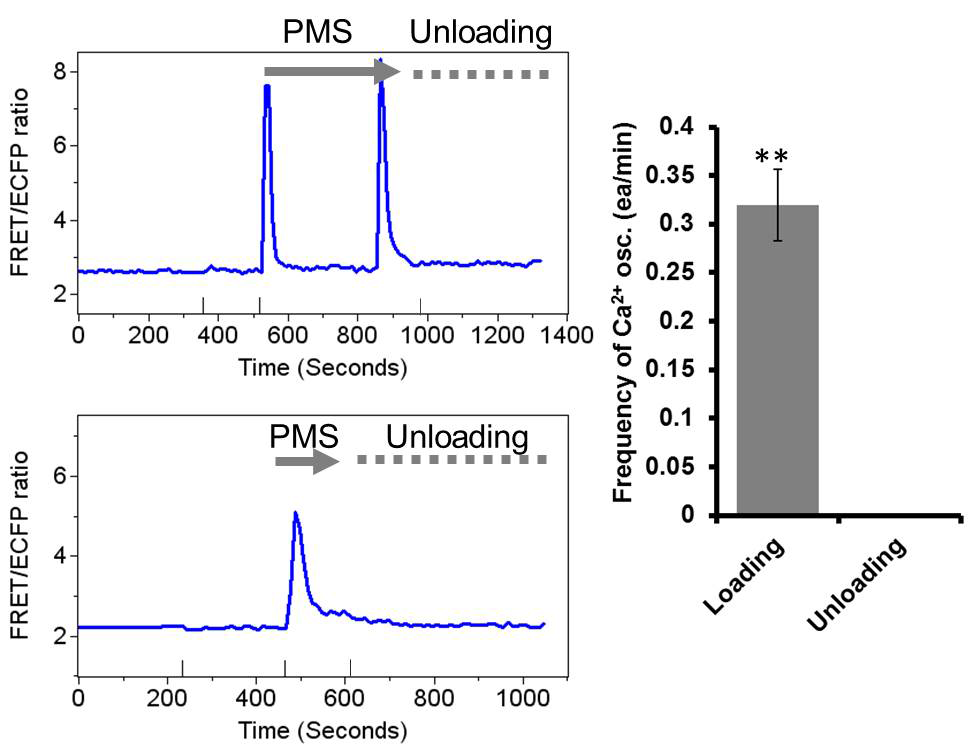

Supplement: Figure S2 — Calcium signals in response to PMS and unloading. hMSCs showed the PMS-induced calcium signals, but when stretch stopped, calcium oscillations disappeared (n = 3, Loading vs Unloading,** P<0.01). (TIF) [file pone.0109378.s002.tif]

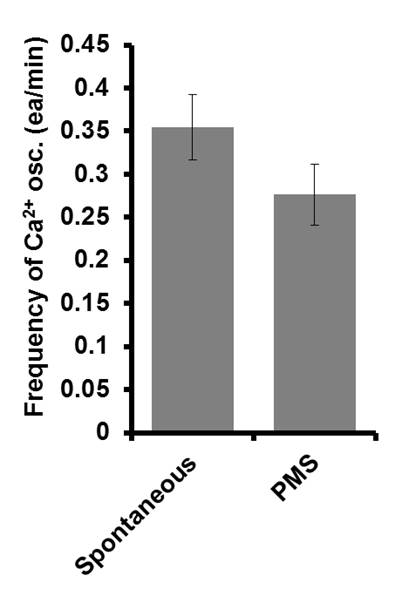

Supplement: Figure S3 — Spontaneous calcium oscillations (n = 33) have similar frequency as PMS-induced calcium oscillations (n = 9). There is no statistical difference. (TIF) [file pone.0109378.s003.tif]
